# Functional Annotation of GWAS Loci Using Public Transcriptome and Epigenome Datasets Reveals Non-Coding Genes and Regulatory Elements Which May Contribute to BMI

**DOI:** 10.3390/ijms27157015

**Published:** 2026-08-04

**Authors:** Chunting Yang, Xiangyuan Yu, Erica L. Kleinbrink, Dale King, Li Chen, Leonard Lipovich

**Affiliations:** 1 Department of Biology, College of Science, Mathematics and Technology, Wenzhou-Kean University, Wenzhou 325035, China; 1235673@wku.edu.cn (C.Y.); dv7925@wayne.edu (L.L.); 2 Wenzhou Municipal Key Laboratory for Applied Biomedical and Biopharmaceutical Informatics, Wenzhou-Kean University, Wenzhou 325035, China; 3 Zhejiang Bioinformatics International Science and Technology Cooperation Center, Wenzhou-Kean University, Wenzhou 325035, China; 4 Department of Biology, New College, University of Toronto, Toronto, ON M5S 1A1, Canada; xiangyuan.yu@mail.utoronto.ca; 5 Quantitative Life Sciences, McGill University, Montreal, QC H3A 0G4, Canada; ekleinbrink.genetics@gmail.com; 6 College of Arts & Sciences, American University of Iraq, Baghdad, Airport Road, Baghdad 10023, Iraq; 7 Center for Molecular Medicine and Genetics, School of Medicine, Wayne State University, Detroit, MI 48202, USA

**Keywords:** body mass index, genome-wide association studies, long non-coding RNA, regulatory variants, epigenomics, obesity genetics, functional annotation

## Abstract

Genome-wide association studies have identified numerous genetic variants statistically significantly associated with body mass index (BMI). However, the functional mechanisms underlying most associations between single nucleotide polymorphisms (SNPs) in non-coding regions and BMI remain poorly understood. Here, we implemented an integrative 7-criterion quantitative scoring system (gene localization, histone modifications, transcription factor binding sites (TFBS), SNP clouds, tissue expression patterns, evolutionary conservation, and COVID-19 associations) to prioritize putative functional loci among 94 BMI-associated SNPs. Six SNPs resided within long non-coding RNA (lncRNA) genes: rs2245368 (exonic, *DTX2P1-UPK3BP1-PMS2P11*), rs2033529 (exonic, *LINC00951*), rs2836754 (intronic, *ETS2-AS1*), rs2815752 (intronic, *LINC02796*), rs17203016 (intronic, *MYOSLID-AS1*), and rs7239883 (intronic, *LINC00907*). We prioritized them because they are located within lncRNA gene bodies and therefore showed stronger functional support compared to other variants which only had non-coding regulatory elements in their vicinity. Notably, rs1928295 exhibited strong GATA2 binding evidence, while rs13201877 had extensive transcription factor occupancy (64 factors). Multiple variants demonstrated putative regulatory potential through epigenomic evidence, including DNase I hypersensitivity and cell-type-specific chromatin accessibility. We show that most BMI risk alleles are not human-specific and are conserved across primates. Our findings suggest putative candidate non-coding regulatory elements in BMI and provide prioritized obesity-associated non-coding variants for functional validations.

## 1. Introduction

Genome-Wide Association Studies (GWAS) are a firmly established approach for identifying genomic regions that are statistically significantly associated with specific complex and quantitative traits as well as with common disease risk factors in large population case–control studies. A hallmark of this approach is the statistical analysis of the genotypes of all known genetic variants, typically single-nucleotide polymorphisms (SNPs), in the genome of every individual in the case and control cohorts. This analysis identifies variants significantly associated with the phenotype of interest and thereby highlights genomic regions that may influence the phenotype through genes or the regulatory sequences located within those regions.

A SNP significantly associated with a trait pinpoints a region, characterized by linkage disequilibrium (LD) with nearby variants, which harbors a functional variant. However, the proportion of significant disease-associated SNPs that localizes to protein-coding gene sequences is well under 5% [1], as is expected, given that protein-coding sequences comprise a mere 1.5% of the human genome [2]. The non-protein-coding nature of the majority of significantly disease-associated variants implies that some of them may reside in long non-coding RNA (lncRNA) genes [3], whereas others may point to enhancers and other non-coding regulatory sequences that modulate the activity of nearby or distant genes [4]. A common methodological constraint of most published GWAS-based functional annotation studies is their emphasis on protein-coding genes nearest to the significant variants, which disregards the functional potential of non-coding RNA (ncRNA) genes that may encompass, or be closer to, those variants [5]. Intergenic variants identified by GWAS are automatically assigned by conventional annotation pipelines to the closest protein-coding gene, frequently perpetuating the incorrect assumption that the closest protein-coding gene, rather than a non-coding gene or regulatory element nearer the actual SNP, is responsible for the phenotype that is significantly associated with that variant. In many cases, especially when the nearest gene is far from the GWAS variant and is separated from the variant by linkage disequilibrium breakpoints, this is likely an incorrect interpretation [6].

The GWAS paradigm has a lengthy history of identifying genetic variants associated with obesity. An in silico strategy has pinpointed non-coding RNA, in particular microRNA (miRNA)-driven, polymorphisms in obesity. Analyzing genetic variation within miRNA binding sites of mRNA target 3′ untranslated regions identified in GWAS highlighted specific miRNA-mRNA interactions potentially relevant to obesity [7].

The Encyclopedia of DNA Elements (ENCODE) Consortium, through its Gencode human gene catalog [8], canvassed all protein-coding and all micro and macro ncRNA genes, and has been central to the discovery of new regulatory mechanisms [9]. Prior to ENCODE, the FANTOM (Functional Annotation of Mammalian cDNA) Consortium determined that mammalian genomes harbor a greater number of non-coding than coding genes [10], many of which are primate-specific [11]. These findings provide a rationale for examining gene structures at human body mass index (BMI)-associated GWAS loci with evolutionary tools capable of highlighting evolutionary novelties. Annotating GWAS loci without restrictive assumptions about conservation can facilitate the discovery of evolutionarily new mechanisms, yield hypotheses for follow-up validation studies, and provide insight into new drivers of old complex traits. Because elevated BMI is closely linked to obesity, diabetes, and broader metabolic dysregulation, these loci may also have implications beyond adiposity. Glucagon-like peptide-1 (GLP1) receptor agonists, the current first-line drugs for diabetes and obesity, have also been shown to delay aging in animal models (Poupon-Bejuit et al. [12]). These findings point to the interplay of diabetes risk and longevity, as well as to unanticipated benefits of established diabetes drugs such as metformin, which attenuates virus-induced cellular senescence (VIS) in COVID-19 patients [13]. Severe COVID-19 outcomes are consistently positively correlated with obesity and elevated BMI [14,15,16]. Therefore, COVID-19 severity serves as an additional correlate and associative measure of BMI-related biology.

Obesity and adiposity are distinct yet interconnected complex traits. Obesity, commonly assessed using BMI, is associated with cardiometabolic disease, premature mortality, dyslipidemia, metabolic syndrome, diabetes, cardiovascular disease, and stroke [17,18,19,20]. There is a substantial genetic component to both obesity and adiposity risk [21]. But lifestyle and socioeconomic factors also shape risk. This condition is a mounting global challenge that constitutes a substantial portion of the worldwide economic and social burden of common non-communicable diseases [22]. Their public health burden continues to rise globally, including in China, India and the United States [22,23,24,25,26,27].

Because high BMI intersects with metabolic dysregulation, diabetes, and severe COVID-19 outcomes, BMI-associated loci may also illuminate broader immune–metabolic interfaces [14,15,16]. This reinforces the need for molecular, mechanism-driven interpretation of obesity-associated variation. GWAS has pinpointed numerous variants significantly associated with BMI. Locke et al. [28] reported 94 BMI-associated variants in 339,244 individuals, and subsequent analyses linked genes near these loci to central nervous system tissues involved in hunger, reward, and energy balance [28,29,30,31]. The same loci were enriched for promoter, histone, and enhancer marks in neuronal tissues, indicating that regulatory mechanisms may contribute substantially to human adiposity [28].

Despite these advances, little is known about the underlying putative functional elements. The overwhelming majority of the significant BMI-associated SNPs identified in the Locke et al. GWAS is in non-coding regions [28]. Of the 94, only two were annotated as missense variants in the exons of protein-coding genes, and an additional one as an exonic synonymous variant. The first two post-genomic decades have revealed that non-protein-coding sequences, including non-coding RNA genes as well as regulatory elements outside of genes, are of seminal regulatory significance [5,32]. Nonetheless, these have never been systematically examined genome-wide in the context of BMI. Our study addresses the potential functional roles of non-coding variants by identifying those that putatively contribute or appear likely to contribute to BMI etiology through an assessment of their regulatory potential within the associated loci.

Here, we undertake a comprehensive examination of the BMI-associated genomic regions from Locke et al. [28], using the UCSC Genome Browser resources [33], ENCODE, the Epigenome Roadmap, GTEx, FANTOM5, and other published transcriptomic, and epigenomic datasets. Our approach emphasizes the transcriptomic and epigenetic properties of heretofore-ignored non-coding sequences that encompass, or positionally precisely overlap, polymorphisms showing statistically significant genetic risk association with BMI, helping to elucidate the biological meaning of those GWAS-pinpointed variants to an extent that reliance on protein-coding genes cannot accomplish.

## 2. Results

### 2.1. Identification of lncRNA-Associated SNPs

In our integrative analysis of 94 BMI-associated SNPs, we identified six as residing within lncRNA genes: rs2245368 and rs2033529 (exonic); as well as rs2836754, rs2815752, rs17203016, and rs7239883 (intronic), sorted in descending order of evaluation scores (Supplementary Table S2). We prioritized these intragenic variants because SNPs located within lncRNA gene bodies may have more direct functional relevance than variants located only near non-coding regulatory elements, consistent with our previous study of lncRNA-associated variants in common metabolic diseases [34]. These six variants were therefore selected for further genomic, epigenomic, transcriptomic, and evolutionary annotation.

### 2.2. Genomic and Epigenomic Annotation of BMI-Associated Genetic Variants

a. SNP localization relative to gene structure

**Figure 1:**
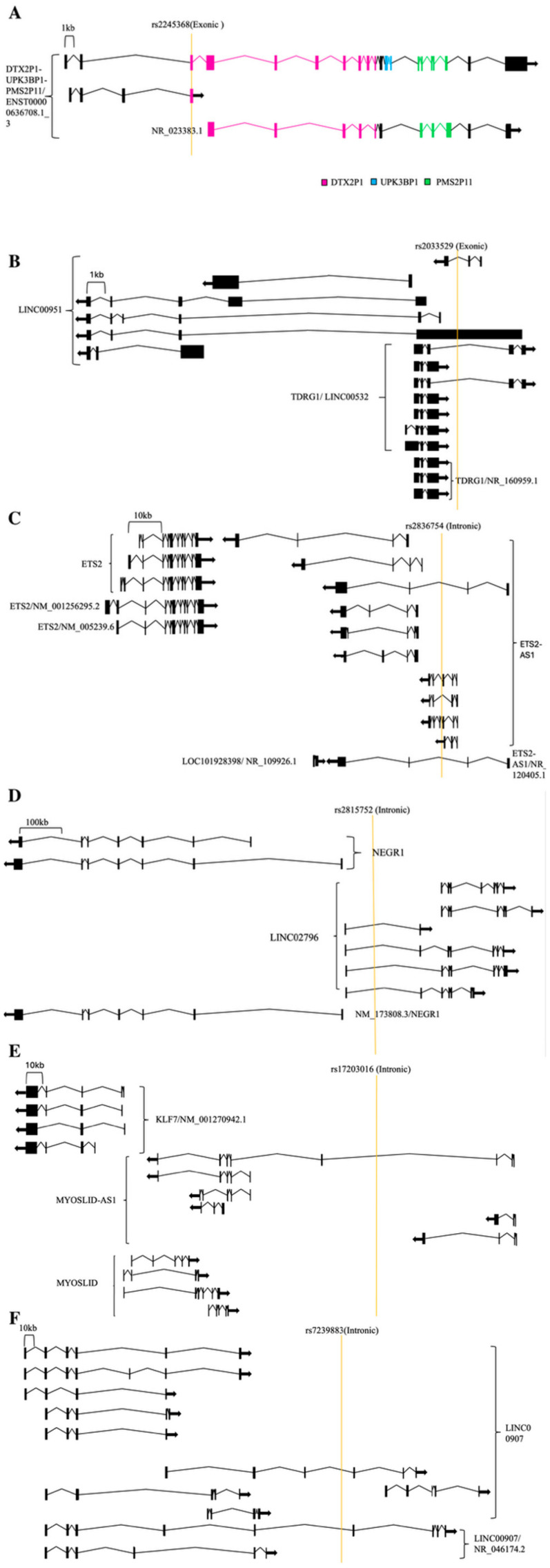
The genomic structure of lncRNA genes that contain six BMI-associated SNPs (rs2245368, rs2033529, rs2815752, rs7239883, rs17203016, and rs2836754), based on GENCODE and NCBI RefSeq reference gene models (hg19). Orange vertical lines indicate the positions of the SNPs. Black boxes represent exons, connecting lines represent introns, and arrows denote transcriptional orientation. Figures not to scale. (**A**) rs2245368 locus: A single RefSeq transcriptional unit (*DTX2P1-UPK3BP1-PMS2P11*, NR_023383.1) is annotated, structurally concordant with the GENCODE model. Pink lines: *DTX2P1*; blue lines: *UPK3BP1*; green lines: *PMS2P11*. (**B**) rs2033529 locus: The SNP is located within the *TDRG1*-*LINC00951* sense–antisense lncRNA gene pair, mapping to an intron of *TDRG1* and an exon of LINC00951. (**C**) rs2836754 locus: The SNP resides in an intron of the lncRNA *ETS2-AS1* (NR_120405.1). Note that *ETS2-AS1* does not overlap with *ETS2* and is not genuinely antisense to it. (**D**) rs2815752 locus: The RefSeq gene *NEGR1* (NM_173808.3) does not encompass the SNP. The SNP is located within LINC02796, which shares a bidirectional promoter with *NEGR1*. (**E**) rs17203016 locus: The SNP is positioned within the lncRNA *MYOSLID-AS1*. *MYOSLID-AS1* is antisense to and partially overlaps with *MYOSLID*, which in turn shares a bidirectional promoter with the protein-coding gene *KLF7*. (**F**) rs7239883 locus: The SNP is located within an intron of the single annotated RefSeq lncRNA gene *LINC00907* (NR_046174.2).

The six lncRNA-intragenic significant BMI-associated SNPs were annotated based on their genomic position, because SNPs in exons of a gene are more likely to point to the direct functional impact of that gene on the phenotype under analysis. The variant rs2245368 was located in an exon of *DTX2P1*. This exon is a part of the intergenically spliced transcriptional unit *DTX2P1-UPK3BP1-PMS2P11*, which is comprised of three transcribed pseudogenes. A large number of SNPs were observed in *DTX2P1-UPK3BP1-PMS2P11* that were mostly associated with BMI and related traits such as body weight and metabolic syndrome, although the relatively low density of and large spacing between the SNPs did not satisfy our definition of a SNP cloud (Figure 1). The BMI-associated SNP rs2033529 was located in an exon of the lncRNA gene *LINC00951* and, due to the multiple overlapping genes at this locus, simultaneously resided in an intron of another lncRNA gene (*TDRG1*/*LINC00532*).

The SNPs rs2836754, rs2815752, rs17203016, and rs7239883 are intronic within the lncRNA genes *ETS2-AS1*, *LINC02796*, *MYOSLID-AS1*, and *LINC00907*, respectively. The functional role of the lncRNA *MYOSLID-AS1* (an uncharacterized transcriptional unit that overlaps another lncRNA gene, *MYOSLID*, in the antisense orientation) remains largely uncharacterized. This lncRNA gene harbors SNPs associated with systolic blood pressure, sex hormone-binding globulin levels, body height, BMI, and metabolic syndrome. The lncRNA *MYOSLID*, the other member of this sense–antisense lncRNA pair, amplifies the vascular smooth muscle differentiation program in a serum response-dependent manner [35].

The lncRNA *ETS2-AS1* contains a cluster of SNPs significantly associated with heel bone mineral density, prostate-specific antigen amount, bone fracture, and BMI. The adjacent *ETS2* gene encodes a protooncogene involved in the regulation of telomerase. Lastly, BMI SNP-containing *LINC02796* and *LINC00907* are novel lncRNAs from high-throughput transcriptome and annotation projects that lack any functional characterization in the literature.

In addition to these six SNPs in lncRNA genes, we considered all SNPs annotated as intronic and intergenic. Among the 94 SNPs, only 3 were located in exons of protein-coding genes, consistent with the known rarity of protein-coding variants in GWAS data [1]. In contrast, 43 SNPs (the prevalent class) were located in the introns of protein-coding genes, while 42 resided in intergenic regions.

b. Epigenomic evidence

Of the 94 loci, 16 had the signal in the Deoxyribonuclease I (DNase I) Hypersensitivity Clusters Track (Supplementary Table S1), a widely used proxy for open-chromatin regulatory elements including promoters, enhancers, and insulators. We also considered promoter and enhancer histone modification signatures. Only one SNP, rs9374842, resided within a peak for H3K4Me1 in ChIP-seq data from normal human embryonic kidney cells, but there was no peak at the same genomic location for H3K27Ac, the other enhancer-associated epigenetic modification. None of these 94 loci had a signal for H3K4Me3 in ENCODE ChIP-seq data, meaning that there are probably no previously uncharacterized promoters in this SNP set. The clear, above-background, visually apparent H3K4Me1 ChIP-seq peak in the ENCODE Regulation track of the UCSC Genome Browser corresponding to this SNP is illustrated in Supplementary Figure S1 as an example of how we implemented visual peak calls.

### 2.3. Transcription Factor Binding Sites (TFBS)

ENCODE TFBS ChIP-seq data showed that, from amongst the 94 loci, 7 had a signal in the ENCODE 338 TFBS track, and 5 of those 7 also had a signal in the ENCODE 161 Consensus TFBS track. All seven SNPs that reside within ENCODE TFBS ChIP-seq signals are intergenic. The variant rs7599312 was informative for two of the seven annotation criteria. In ENCODE ChIP-seq TFBS data, two transcription factors (CTCF and RAD21) bind to the immediate genomic DNA region containing this SNP, and one transcription factor (CTCF) was also identified in the ENCODE 161 Consensus TFBS track (Supplementary Table S1). The variant rs2176040 was, similarly, informative for two of the seven features, but only one transcription factor (FOXP1) binds the immediate 501-bp genomic DNA interval containing this SNP in the ENCODE 338 dataset, with no transcription factor binding identified in the ENCODE 161 Consensus track. FOXP1 controls adipocyte differentiation [36], suggesting a possible direct relationship of this binding event to BMI. In contrast, rs13201877 was also informative for only two of the seven features, but notably, numerous transcription factors bound its genomic interval in ENCODE, strongly suggesting a regulatory role or a potential super-enhancer signature, and multiple transcription factors were also identified at this locus in the ENCODE 161 Consensus TFBS track, further supporting its regulatory potential (Supplementary Table S1). The variant rs1928295 had one transcription factor (GATA2) binding its immediate genomic interval in ENCODE ChIP-seq, and GATA2 was also identified in the ENCODE 161 Consensus TFBS track; GATA2 is an immunomodulator implicated in adipocyte differentiation and obesity-related pathways (Supplementary Table S1) [26,37]. The variant rs3888190 had multiple transcription factors binding its genomic interval in the ENCODE 338 dataset, and one transcription factor (MAFK) identified in the ENCODE 161 Consensus TFBS track (Supplementary Table S1). Similarly, rs7164727 has multiple transcription factors binding within its genomic interval in the ENCODE 338 dataset, and one transcription factor, TEAD4, identified in the ENCODE 161 Consensus TFBS track; TEAD4 has been reported to repress adipogenesis (Supplementary Table S1) [26].

### 2.4. SNP Clouds

Among the 94 BMI-associated index SNPs, seven had at least one additional significant GWAS variant within 501 bp: rs2815752, rs1514175, rs2867125, rs205262, rs1558902, rs9914578, and rs2075650. However, three of these loci did not meet our definition of a BMI-relevant SNP cloud because their neighboring variants were associated with phenotypes not directly related to BMI. Specifically, rs939582 and rs2867124 near rs2867125 were associated with cigarette smoking; rs2744977 near rs205262 was associated with male-pattern baldness; and rs9914577, rs71902577, and rs539275859 near rs9914578 were associated with immune cell counts. Therefore, four index SNPs met our predefined criterion for inclusion in a BMI-relevant SNP cloud: rs2815752, rs1514175, rs1558902, and rs2075650.

Among the six lncRNA-intragenic SNPs, only rs2815752 met our definition of residing in a SNP cloud. The other SNPs near this index SNP include rs2815753, which is associated with body surface area, and rs2568960, which is associated with depression as well as with insomnia and other sleep disturbances potentially contributing to anxiety, weight gain, diabetes, and cardiometabolic disease progression. This suggests potential for the lncRNA gene *LINC02796*, which harbors this SNP, to be a direct susceptibility determinant for those conditions. Histone modification patterns and TFBS occupancy in the genomic region containing this SNP indicate tissue-specific regulatory activity, particularly in skeletal muscle, liver, and brain.

Select intronic SNPs in protein-coding genes, such as *ZC3H4* (rs3810291), indicate putative functional potential through regulation by antisense RNAs and super-enhancers, linking transcriptional control to adipogenesis and inflammation [38]. The 48 SNPs that are located in coding-gene introns and in intergenic regions but have compelling evidence of transcription factor binding and/or histone modifications in ChIP-seq data may influence adiposity by modulating chromatin accessibility, TF binding, and gene expression, collectively shaping gene regulatory networks that impact metabolic traits. The prevalence of histone modifications and TFBS occupancy in BMI-related tissues reinforces the functional significance of these variants and supports their prioritization for experimental validation.

### 2.5. Tissue Expression and Cell Type Specificity

The lncRNAs containing intragenic BMI SNPs are, intriguingly, mostly expressed in tissues related to BMI. The SNP rs2245368 is located in an exon of the intergenically spliced transcribed pseudogene *DTX2P1-UPK3BP1-PMS2P11*, which is expressed in metabolic tissues and organs such as fat, small intestine, and stomach, potentially indicative of a connection with BMI regulation. In GTEx RNA-seq data (Release V8), *DTX2P1-UPK3BP1-PMS2P11* was also expressed in tissues related to BMI, such as fat, bladder, small intestine, spleen, and stomach. The expression profile of the intergenically spliced *DTX2P1-UPK3BP1-PMS2P11* TU, but not of any of its three component pseudogenes, is suggestive of BMI regulation relevance.

The BMI-associated SNP rs2033529 resides in an exon of the lncRNA *LINC00951*, but its GTEx coverage did not include BMI-relevant tissues or organs, a common expression scenario we witnessed for other SNPs as well. To address this, we established a new scoring criterion: in cases where GTEx is unavailable or uninformative, we examined the EST (Expressed Sequence Tag) track and full-length cDNA (Genbank mRNA) tracks of the UCSC Genome Browser for evidence of transcripts demonstrating overall genomic footprint matching, including exonic overlap and directional consistency, to the lncRNAs’ RefSeq and Gencode reference transcript models. A score of 0.2 was assigned if there was evidence of overlap with either ESTs or cDNAs, while fulfillment of both criteria resulted in a score of 0.5. For *LINC00951*, ESTs and cDNA libraries yielded a 0.5 score because of their concordance. The SNP rs17203016 resides within *MYOSLID-AS1*, a testis-specific lncRNA, and thanks to EST support, was scored 0.2. The SNP rs2836754 is intronic to *ETS2-AS1*, which has liver-specific expression and links to metabolic and bone traits, and has mRNA support but no ESTs (0.2). The SNP rs2815752 is within *LINC02796*, near a SNP cloud containing additional variants significantly associated with insomnia and obesity (the former being a well-known risk factor for the latter), and has cDNA and EST support, but lacks GTEx data (score 0.5). Finally, rs7239883 in *LINC00907* shows liver-specific expression and strong conservation across vertebrates and has only mRNA support (score 0.2). In total, of the original 94 SNPs, 52 SNPs’ nearest or overlapping genes have available GTEx data.

GTEx lacks tissue expression data for certain SNPs’ nearest or overlapping genes. To validate the tissue-specific expression signals inferred from EST and cDNA datasets, we examined whether additional relevant tissues, not captured by previous approaches, could be identified. Among all 94 SNPs, 59 SNPs’ nearest or overlapping genes have FANTOM Consortium promoterome data in the ZENBU Browser [39,40], and most of their FANTOM expression profiles were consistent with those inferred from GTEx, EST, and cDNA data wherever those were available.

In total, 59 SNP-containing genes and TUs were analyzed in FANTOM5 Cap Analysis of Gene Expression transcription start site activity data in approximately 1000 cell lines and tissue types (“human hg19 promoterome”) to characterize their promoter activity across a diverse range of tissue types, developmental stages, and cell lineages, including both normal and cancerous samples. It is noteworthy that 9 promoters of genes nearest to or overlapping BMI SNPs exhibited high expression in adipose tissue cells, including mature adipocytes and preadipocytes, which strongly implicates these genes in obesity and BMI regulation, validating our approach of searching for the overlapping or nearest genes and TUs of BMI-associated SNPs and considering these as direct causal candidates regardless of their protein-coding capacity. The screen also revealed that brain regions, specifically substantia nigra—adult, donor10196 (6 occurrences) and optic nerve, donor1 (5 occurrences), along with hematopoietic cell lines, common myeloid progenitors (CMP), donor and neutrophil (PMN), donor1 (each with 4 occurrences), were seen most frequently in the transcriptome data for these BMI SNPs’ nearest genes, appearing more than three times. Substantia nigra is central to drug abuse and dopamine pathways that may be common to drug and food addiction.

For the intergenically spliced transcribed pseudogene which harbors the SNP rs2245368, FANTOM data from the 5′-most TSS indicates expression in epithelial and secretory tissues, including bronchial, tracheal, esophageal and corneal epithelial cells, as well as lipid cells and eosinophils. This extensive distribution highlights the potential role of this variant in maintaining the integrity of the epithelial barrier and the immune interaction on the mucosal surface. Several of these tissues, such as the trachea and esophagus, are also supported by expression data from GTEx (esophagus, salivary gland) and EST (trachea) datasets, indicating independent validation of this lncRNA’s top-expressor tissues with cross-platform consistency.

The SNP rs2033529 resides in a sense–antisense pair of lncRNA genes: *TDRG1—LINC00951*. There is no FANTOM5 expression data for *TDGR1*. In the FANTOM5 promoterome, *LINC00951* was expressed in adult testis in multiple samples and in various cancer-derived cell lines, including pagetoid sarcoma, adult T-cell leukemia (ATN-1), chondrosarcoma (SW 1353), and chronic myeloid leukemia (K562), which are confirmed by ENCODE data. This expression profile indicates potential roles in germline and malignant environments and may reflect the regulatory flexibility of proliferation and differentiation-related pathways. Importantly, GTEx, EST and cDNA data all supported expression in the testis, again providing strong cross-validation.

The FANTOM5 promoterome of *ETS2-AS1*, the lncRNA gene that contains the significant BMI-associated SNP rs2836754, shows that it is widely expressed in mesenchymal cell types, including liver, umbilical and adipose-derived mesenchymal stem cells, as well as differentiated cell populations such as subcutaneous adipocytes, aortic smooth muscle cells and cardiac fibroblasts. Additional activity was detected in myeloid progenitor cells, granulocyte–macrophage progenitor cells and chondrocytes, highlighting their potential role in matrix and hematopoietic differentiation [41]. GTEx supports liver expression of this lncRNA, while cDNA data shows that it is also present in the brain; both are relevant to BMI, the former metabolically and the latter through behavioral aspects of addiction to high-calorie diets.

The variant rs2815752 belongs to the gene pair *LINC02796-NEGR1*, which has a shared tissue expression, due to its shared bidirectional promoter. FANTOM data shows transcriptional activity in connective tissue and structural cell types, including Achilles tendon, nucleus pulposus, lymph fibroblasts, aortic endothelial cells and chorionic cells, suggesting a role in musculoskeletal and vascular regulation. Other signals were observed in cancer cell lines such as DJM-1 (malignant papillary cyst), PC-14 (lung adenocarcinoma), ECC4 (gastrointestinal cancer), and hematopoietic malignancies (T-CLL series SKW-3), emphasizing the possible association with proliferation dysregulation. EST/cDNA data indicated expression in normal embryonic tissues as well.

Transcriptome data for the lncRNA *MYOSLID-AS1* (containing the significant BMI-associated SNP rs17203016) highlights the hematopoietic and immune systems. FANTOM data shows that its transcriptional activity spans multiple myeloid lines, including eosinophils, neutrophils, mast cells, promyelocytes/myeloid cells, and common myeloid progenitor cells, indicating a possible role in granulocyte differentiation and innate immune regulation. Consistent with this, GTEx detected expression of this lncRNA in whole blood.

The expression profile of *LINC00907*, containing the BMI variant rs7239883, points mainly to cancer. FANTOM data revealed transcriptional activity in retinoblastoma, medulloblastoma, and small cell lung cancer cell lines, highlighting neuroectoderm and neuroendocrine tumors.

FANTOM5 promoterome data demonstrates that BMI SNP-containing lncRNAs and transcriptional units often exhibit distinct expression patterns in metabolic and regulatory tissues, such as adipose tissue, liver, and specific brain regions. These results suggest that a subset of these lncRNAs is expressed in tissues relevant to BMI regulation, supporting their potential roles as functional mediators of obesity-associated genetic risk.

Taken together, FANTOM5 and GTEx results demonstrate that the majority of our prioritized lncRNAs are expressed in metabolically relevant tissues and immune-related biological contexts. By integrating these datasets, our approach accounts for cell-type specificity across multiple BMI-relevant tissues, including adipose, liver, brain, and the immune system, thereby supporting the potential roles of these non-coding transcripts as functional mediators of obesity-associated genetic risk.

### 2.6. Evolutionary Conservation

The six lncRNA-intragenic SNPs exhibited varying degrees of evolutionary conservation among species. At the SNP rs2245368, the human C allele was observed in all reference assemblies of non-human primates that possess the orthologous sequence fragment. At this locus, T is the risk allele in humans, with its prevalence varying by population. If a human disease-associated allele of a variant was not found as the reference assembly in any closely related species, in a locus that is highly conserved, then the allele is more likely to be disease-causative, and the SNP may contribute to the susceptibility to the associated disease or trait [42]. By this logic, the apparently human-specific T allele at this locus is a disease candidate. However, Supplementary Table S1 shows that the T allele is also present in both Neanderthals and Denisovans, indicating that it is not modern-human-specific but instead arose earlier in hominin evolution.

For instance, the rs9540493 locus exhibits a high degree of evolutionary conservation, with the ancestral A allele—the risk allele for high BMI in modern humans—being fixed across Neandertals, Denisovans, and both primate and non-primate mammalian species. We conclude that the ancestral-state variant, which presumably did not cause disease in the common ancestor, any nonhuman primate species, or ancient humans (Denisovans and Neanderthals), is now associated with disease risk in modern humans.

At rs2033529, in most non-human primates, the reference assembly base is A, whereas in humans, the locus harbors an A/G polymorphism, but the risk allele is not consistently reported, as approximately equal numbers of Genome-Wide Association Studies indicate the disease risk allele to be A or G. Both alleles are observed in Neanderthals and Denisovans, demonstrating that this polymorphism was already established before the divergence of modern and archaic human lineages (Supplementary Table S1).

For rs17203016, in all reference assemblies of non-human primates, the reference base is A, while in humans, the locus is A/G polymorphic, but the risk allele is also not consistently reported in GWAS. We designated G as the risk allele based on the number of distinct published GWAS stating it as such (per the NHGRI-EBI GWAS Catalog track of the UCSC Genome Browser), although the number of GWAS supporting A as the risk allele was only one fewer. The presence of both alleles in Neanderthal and Denisovan genomes suggests that this variation reflects an ancestral hominin polymorphism rather than a modern human-specific mutation (Supplementary Table S1).

Most non-human primates have C as the reference base at the location orthologous to the SNP rs2836754, where a C/T variant resides in humans, and the risk allele differs between populations. Supplementary Table S1 indicates that the T allele is also observed in Neanderthals and Denisovans, indicating that it arose prior to the divergence of modern and archaic humans.

For the SNP rs2815752, all non-human primates exhibit G as the reference base at the orthologous genomic location, whereas humans have an A/G variant, with A as the major risk allele. Consequently, A is likely the pathogenic allele, based on published assumptions [42]. This A allele is also present in Neanderthals and Denisovans, indicating that it is not unique to modern humans (Supplementary Table S1).

Finally, for the SNP rs7239883, all 100 analyzed vertebrate sequences exhibit a G as the reference base at this highly conserved locus. In humans, the variant is an A/G polymorphism, but the risk allele is not uniformly defined as it varies by population. It is not uncommon for risk alleles to lack uniform definition, because of subpopulation specificity of genetic risk as well as other phenotypes or diseases linked to the same variant in the index population or other populations. Supplementary Table S1 shows that both alleles are present in Neanderthals and Denisovans, indicating that this polymorphism is shared across hominin lineages.

In Supplementary Table S1, we list all the sample information of these 94 SNPs from UCSC’s NHGRI-EBI GWAS track, including ethnicity and the corresponding risk allele, and select the risk allele with the largest number of samples as the major risk allele, wherever that is possible. We analyzed the conservation of each BMI disease risk allele in modern humans, Neanderthals, Denisovans, non-human primates, and non-primates. The results indicated that the alleles of these disease-related SNPs were highly conserved across these organisms, because 40 of them have the same alleles in modern humans, Neanderthals, Denisovans, non-human primates, and non-primates. In total, 75 SNPs had their human risk allele as the reference genome assembly base in at least some of the non-primates. For ten SNPs, the human risk allele was only present in modern humans. Four SNPs had the human risk allele as the reference assembly sequence in at least some nonhuman primates (but not in any non-primates). A further five SNPs had the human risk allele detectable in Neanderthals and Denisovans, but not in any nonhuman species extant today, indicating that the appearance of the risk allele pre-dated the origin of ancient humans, and that the risk allele was not unique in modern humans in these cases.

### 2.7. COVID-19 Associations

The six lncRNA-intragenic SNPs exhibited varying extents of overlap with COVID-19 GWAS v4 tracks, suggesting potential pleiotropic effects related to immune or metabolic regulation. The SNPs rs2245368, rs17203016, and rs2815752 each showed protective (negative) associations with COVID-19 severity and hospitalization in 3 out of 4 sub-tracks and harmful/positive associations in 1, indicating a possible dual role in host susceptibility. The variants rs2033529 and rs2836754 also demonstrated mixed signals across the tracks. In contrast, rs7239883 was not associated with COVID-19 severity. These overlaps point to a shared regulatory landscape between metabolic traits and immune response to viral infection. Non-lncRNA BMI SNPs also contributed to COVID-19 susceptibility. A prime example is rs1928295, predicted to play a key role in the regulation of GATA2. This is supported by clinical evidence, as GATA2 haploinsufficiency has been implicated as a potential underlying factor in critical COVID-19 pneumonia [43]. Among the 94 SNPs, only 13 had no COVID data, while most of the SNPs with four tracks had protective or negative results. This is surprising because high BMI is a positive correlate of COVID-19 severity.

### 2.8. High-Scoring Intergenic and Intronic SNPs

Beyond SNPs residing in lncRNA genes, our analysis identified a subset of high-scoring SNPs of particular interest within intergenic and intronic regions. For instance, the intronic SNP rs1928295 (score: 3) presents compelling evidence of residing within a functional regulatory element. ENCODE DNase I hypersensitivity data reveals that this locus is a site of open chromatin in 3 of 125 cell types, a restricted pattern of chromatin accessibility indicating potential cell-type specificity.

Further support for its regulatory function is provided by TFBS predictions. The ENCODE 338 TFBS track identified nine TFs with predicted binding at this locus: GATA2 (Cluster Score: 869/1000), CTCF (196/1000), RCOR1 (378/1000), GATA3 (552/1000), RFX5 (213/1000), RFX1 (33/1000), MXI1 (250/1000), EP300 (249/1000), and RAD21 (65/1000). To enhance confidence in these predictions, we cross-referenced this locus with the more stringent ENCODE 161 Consensus TFBS track. This consensus analysis corroborates the binding of three key TFs: GATA2 (Cluster Score: 1000/1000), CTCF (159/1000), and GATA3 (363/1000). The high-confidence DNase I hypersensitive site (DHS) that overlaps with TFBS predictions, and the high and concordant scores for GATA2 across both the ENCODE 338 (869) and the consensus 161 (1000) tracks, suggest that rs1928295 resides within an active, cell-type-specific regulatory element that is functionally bound by the GATA2 transcription factor. As GATA2 suppresses adipogenesis while activating vascular cells, its inhibition may represent as a novel strategy to reduce obesity-induced inflammation and avert downstream complications such as diabetes and plaque rupture [44].

The intergenic SNP rs7164727 (score 3) falls within clusters in 4–8 of 125 cell types in ENCODE DNase I hypersensitivity (open chromatin) data. The ENCODE 338 TFBS ChIP-seq track demonstrates the binding of nine TFs at this locus, including ELF1 (ENCODE TFBS cluster score 61/1000), FOS (181/1000), GATA3 (741/1000), CREB1 (234/1000), and NFE2L2 (634/1000). Additionally, the ENCODE 161 Consensus TFBS ChIP-seq track indicates TEAD4 binding (290/1000). Among these, GATA3, NFE2L2, and TEAD4 exhibit the highest scores.

NFE2L2 is a major anti-inflammatory marker, and a variant in this gene modifies the adverse impact of obesity on heart rate variability [45]. Being overweight is associated with elevated inflammatory markers and autonomous nervous system imbalance. In adipogenesis, multiple transcription factors cooperatively regulate the expression of the PPARG2 gene, a critical step in adipocyte differentiation. Specifically, TEAD4 enhances the occupancy of glucocorticoid receptors (GR) and C/EBPβ at the hotspot region of the PPARG2 promoter, thereby promoting key regulatory events in early adipogenesis [46].

### 2.9. CeRNA Network Analysis

Within the well-established competing endogenous RNA (ceRNA) regulatory framework [47], lncRNAs bind specific miRNAs (through complementary base pairing) to prevent them from downregulating their cognate mRNA targets. Since we prioritized several lncRNA genes that had been previously overlooked as potential contributors to BMI etiology despite containing GWAS significant BMI variants, we tested the hypothesis that these lncRNAs may act as ceRNAs. This would offer a plausible mechanistic explanation for how these non-coding variants exert trans-regulatory effects on distal BMI-related genes.

We investigated the ceRNA crosstalk between SNP-associated lncRNAs and protein-coding genes. Based on predictions from LncBase v3 and TargetScan, we initially identified a broad set of lncRNA–miRNA–mRNA triplets (Supplementary Tables S3–S5). To characterize the broader regulatory landscape, we first constructed a global ceRNA network using Bgee expression data, retaining components only if their expression scores exceeded 60 in at least one common human tissue. This exploratory model yielded 109 tissue-supported triplets (Supplementary Figure S1).

**Figure 2:**
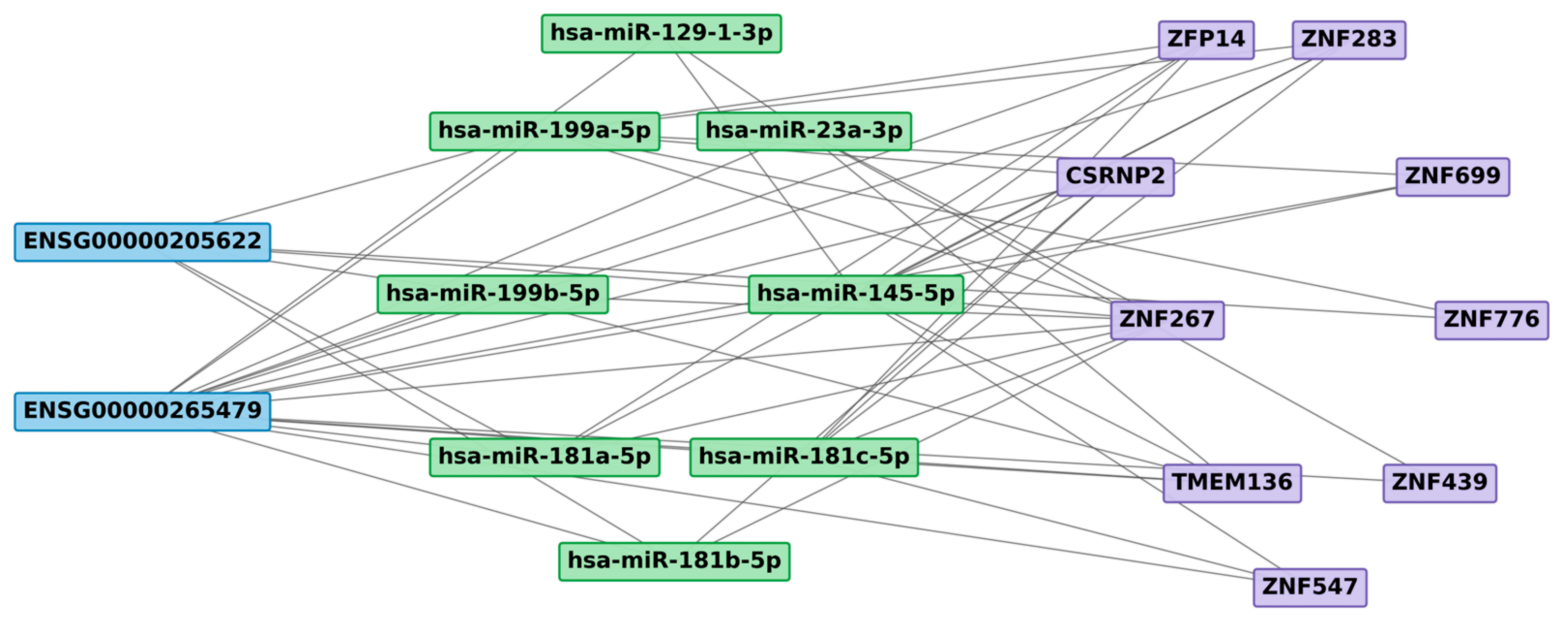
Tissue-supported regulatory interactions of prioritized lncRNAs. The network illustrates high-confidence ceRNA axes filtered by GTEx expression data (TPM ≥ 1) to ensure co-expression of lncRNAs and mRNAs within the same human tissue. LncRNAs (blue nodes), miRNAs (green nodes), and mRNAs (purple nodes) form potential regulatory pathways through which prioritized SNPs may influence BMI-related molecular networks.

We then focused on a streamlined set of high-confidence interactions supported by GTEx expression data (Figure 2). In this refined network, lncRNAs and mRNAs were preserved only when transcript expression reached TPM ≥ 1 within the same human tissue to ensure consistent co-expression. This more conservative approach identified a focused set of 21 triplets involving 2 lncRNAs, 8 miRNAs, and 9 mRNAs. A comparison of the two filtering platforms revealed that 17 lncRNA–miRNA–mRNA triplets were consistently preserved in both the Bgee- and GTEx-supported networks (Supplementary Table S6).

Within this prioritized network, hsa-miR-181a-5p, which interacts with two of our prioritized lncRNAs (ENSG00000265479 and ENSG00000205622), stood out by interacting with three mRNAs (*ZFP14*, *ZNF283*, and *CSRNP2*), suggesting a potential regulatory influence on these targets. Notably, the prioritized lncRNA gene ENSG00000265479 (the DTX2P1 transcribed pseudogene), containing the BMI-associated variant rs2245368, exhibited the strongest centrality, participating in 15 interactions. This may point to the function of this transcribed pseudogene as a microRNA sponge that deflects microRNAs from the mRNAs of its parental genes and suggests its potential role as a functional ceRNA hub within BMI-relevant tissues, providing a specific candidate testable mechanistic pathway for how this intra-lncRNA variant may influence distal gene expression.

## 3. Discussion

Here, we performed an integrative exploratory annotation of 94 BMI-associated SNPs from Locke et al. [28], in order to identify variants with direct functional potential, particularly in non-coding regions. The principal result is that several BMI-associated variants are more informatively interpreted through lncRNA genes, local regulatory evidence, tissue-specific expression, and evolutionary context than through nearest protein-coding genes alone.

The majority of published annotations of statistically significant disease-associated genetic variants from GWAS have focused on protein-coding genes nearest to the variants, hence often assigning intergenic SNPs to the nearest coding gene, which may misrepresent functional relevance. In contrast, our approach emphasizes functional signals (transcriptomic and epigenetic), as well as evidence for non-coding RNA transcription, directly at, or overlapping, the actual genomic locations of SNPs. By utilizing contemporary gene catalogs and regulatory element data that were not available when these loci were first identified, we systematically determined the functional potential of each variant. We performed a functional placement of significant BMI-associated variants within specific genomic features, such as lncRNA genes and non-coding regulatory elements, which provides a more precise perspective on their putative regulatory roles.

Gene body localization of a disease-associated genetic variant should not be interpreted as inherently stronger than localization within a non-coding regulatory element. A well-established precedent of this paradigm is the FTO obesity locus, where the intronic variant rs1421085 disrupts ARID5B repressor binding and alters a distal enhancer circuit regulating IRX3 and IRX5 during adipocyte differentiation [48]. This example illustrates why GWAS loci must be evaluated for distal regulatory effects and motivated our inclusion of histone modification and TFBS annotations. In our dataset, an lncRNA gene body variant is easier to connect to a named transcript, whereas the target gene of a putative regulatory element remains unknown and the variant may act in cis, in trans, or across a long genomic distance. That difference reflects better annotation availability in genic loci and the inherent inability to connect a putative enhancer to a target gene without functional experiments; it does not construe independent evidence that lncRNA-associated variants are more causal.

Our findings align with prior observations that most GWAS variants for complex traits are intergenic or intronic [1,49], and support the emerging view that non-coding RNAs, especially lncRNAs, are critical regulators of obesity-related pathways. We identified the lncRNAs *MYOSLID-AS1* (matches 3 of our 7 annotation criteria) and *DTX2P1-UPK3BP1-PMS2P11* (4 out of 7 features) as high-priority candidates for direct causal roles in BMI, as well as in adiposity, metabolism, and postmenopausal osteoporosis [50]. These results build on and refine previous GWAS annotations [5,7,28] by emphasizing the putative functional relevance of non-coding RNAs rather than merely reflecting LD with nearby protein-coding genes.

Several high-scoring BMI SNPs appear to intersect with immune-related pathways, highlighting potential pleiotropy. For instance, rs2245368 and rs2815752 may contribute to both metabolic regulation and immune function, suggesting shared molecular mechanisms underlying obesity, metabolic syndrome, and susceptibility to severe outcomes from viral infections such as COVID-19. rs2245368 resides in the pseudogene *DTX2P1-UPK3BP1-PMS2P11*. Pseudogene-derived lncRNAs are known to regulate inflammatory gene networks and adipogenesis. Therefore, rs2245368 may affect metabolic and immune pathways through cis or trans regulatory effects [51]. According to GeneCards (17 July 2025; GC07P076959; GIFtS: 19), the most significant TFBS at the *DTX2P1-UPK3BP1-PMS2P11* promoter are for AP-4, ARP-1, Brachyury, C/EBPalpha, Evi-1, GATA-1, GATA-2, GATA-3, HOXA5, and Max1.

We analyzed CAGE (Cap Analysis of Gene Expression) RNA sequencing data from the FANTOM5 Consortium’s expression dataset of approximately 1000 human cell and tissue types, via the ZENBU transcriptome browser [40]. We did this for all known genes and for all Genbank mRNA- and/or EST-supported transcriptional units that harbored SNPs from the original list of 94. For rs2245368, FANTOM5 data suggests robust transcription (of the intergenically spliced triple pseudogene harboring this SNP) in several epithelial and immune cell types. If rs2245368 resides within cell-type-specific regulatory elements—such as enhancers or promoters active in these cell types—it may influence local gene regulation in a context-dependent manner.

GWAS results, taken alone, inherently overlook tissue-specific regulatory effects. Hence, it is important to assess the expression profile of SNP-harboring coding and non-coding genes with encyclopedic transcriptome reference resources such as GTex and FANTOM: expression in cell or tissue types relevant to the phenotype that the SNP is significantly associated with, in this case, BMI, adds to evidence for the direct functional importance of the SNP. The variant rs2815752 is significantly associated with obesity and related anthropometric and metabolic indices [52]. In addition, rs2815752 is intronic to the gene *NEGR1*, which links metabolic regulation to immune signaling: *NEGR1* modulates IL-6 trans-signaling, and loss of *NEGR1* in mice alters adiposity and hepatic lipid accumulation, providing a direct molecular path from a BMI-associated variant to inflammatory and metabolic phenotypes [53,54]. This intersection underscores the broader regulatory impact of non-coding variants and their potential involvement in diverse biological processes beyond adiposity. The GWAS COVID-19 v4 track of the UCSC Genome Browser complements our six other scoring criteria, offering additional signals that may uncover novel mechanistic links. Patients with type-2 diabetes and obesity had significantly higher risks of hospitalization and progression to severe COVID-19, suggesting that integrating metabolic disease context enhances our understanding of host susceptibility [55].

The widespread conservation of many BMI-associated alleles across primates suggests that these variants are often not recent human-specific changes, but instead represent older allelic states embedded in long-standing metabolic or regulatory pathways. By investigating the evolutionary age and diversification of risk alleles—including comparisons between primates, ancient humans, and modern humans—we provide a historical functional context for these variants. However, under modern conditions characterized by calorie-rich diets and sedentary lifestyles, these same variants may predispose individuals to obesity and metabolic disorders. A “thrifty” missense variant in *CREBRF* (rs373863828) increases BMI while reducing diabetes risk in Samoan populations, exemplifying how adaptive variants can have maladaptive effects in contemporary settings [56]. Evolutionary pressures have shaped genetic variants that were once beneficial but now contribute to disease susceptibility due to a mismatch between ancestral selection pressures and modern environments [57].

Our results indicate that BMI-associated variants do not all follow the same evolutionary pattern. The “thrifty gene” hypothesis remains a useful evolutionary framework for considering BMI- and obesity-associated traits [58], but our data suggest that the underlying genetic architecture is unlikely to be explained by deeply conserved mechanisms alone. The evolutionary conservation patterns of the annotated BMI-associated loci are heterogeneous (Supplementary Table S1). For example, rs2836754 in *LOC400867* shows comparatively deep conservation, with all key gene structure elements retained as far as mouse. By contrast, several prioritized non-coding loci have shallower conservation boundaries. The lncRNA gene containing rs7239883 (*LINC00907*) is conserved only within primates, while the novel transcriptional units associated with rs2033529 and rs17203016 show divergence within primates, and between primates and nonprimate mammals, respectively. On a more distant scale, the pseudogene transcriptional unit harboring rs2245368 retains most of its key gene structure elements—splice sites and polyadenylation signal—throughout placental mammals. These findings suggest that BMI-associated loci may include both relatively ancient conserved components and more recently evolved, lineage-specific non-coding regulatory structures. Accordingly, our results support a more nuanced interpretation of evolutionary models such as the thrifty gene hypothesis [59].

The evolutionary age and stability of these alleles across different lineages implies that these allelic states, which are currently disadvantageous because they may contribute to high BMI in modern humans, may have provided adaptive advantages in the ancestral environment, such as promoting effective energy storage during periods of food shortage, which is consistent with the “thrifty gene” hypothesis [60]. Secondly, because these alleles are associated with obesity and related metabolic disorders, their persistence in modern humans highlights the evolutionary mismatch between ancient selective stress and contemporary lifestyles. Finally, certain risk alleles are conserved in both modern and ancient humans (Neanderthals and Denisovans). This finding highlights their potential role in basic metabolic pathways and reveals non-coding regulatory elements that may contribute to BMI, which should promote further research on whether these risk alleles were advantageous in ancient humans.

Emerging mechanistic studies suggest that lncRNAs exert regulatory effects in metabolic tissues not merely as transcriptional modulators, but as active participants in post-transcriptional circuitry, through competitive endogenous RNA (ceRNA) mechanisms [61]. In this model, lncRNAs sequester specific miRNAs based on sequence complementarity, thereby relieving repression of miRNA-targeted mRNAs and enabling coordinated regulation of functional gene modules. Such ceRNA dynamics are increasingly recognized as important regulators of adipocyte differentiation, mitochondrial metabolism, and inflammatory signaling within metabolically active tissues [62]. Here, the integration of experimentally supported lncRNA–miRNA interactions with TargetScan-derived miRNA–mRNA pairs, followed by stringent tissue co-expression filtering across Bgee and GTEx, revealed 17 co-expressed lncRNA–miRNA–mRNA triplets. Mechanistically, the prominence of hsa-miR-181a-5p—a miRNA family known to regulate immune–metabolic transitions, adipocyte lineage commitment, and cytokine-mediated metabolic stress responses [63,64]—suggests that ceRNA interactions may operate at key nodes linking inflammation and energy metabolism. Likewise, ENSG00000265479, which exhibited the highest centrality among lncRNAs, may function as a miRNA sponge that modulates the availability of miR-181 family members across relevant tissues. Downstream, the repeated targeting of zinc-finger transcriptional regulators such as *ZFP14* and *ZNF283* further supports a model in which ceRNA interactions fine-tune chromatin-associated gene expression programs essential for metabolic adaptation. Collectively, these findings highlight a potential lncRNA–miR-181–ZNF regulatory axis, implicating a testable mechanistic link between non-coding RNA dysregulation and BMI-associated metabolic phenotypes.

We present a multidimensional manual annotation using diverse public experimentally derived genomic datasets, including those from the ENCODE Consortium, expression data from Genbank cDNA and EST resources, as well as the FANTOM Consortium, allowing accurate definition of transcribed non-coding and regulatory elements with a consistent and reproducible annotation protocol.

Our scoring system assigns equal weight to each annotation criterion. The seven annotation dimensions represent heterogeneous evidence and are hence unlikely to have equal biological importance. However, no validated benchmark currently provides defensible relative weights for these disparate annotation classes. We therefore retained a simple equal-point additive scheme to make the exploratory prioritization transparent and consistent and to avoid introducing unvalidated differential weights; the equal numerical contributions of each of the seven criteria should not be interpreted as equal strengths of biological importance. The scoring thresholds were not optimized against experimentally validated causal variants, because the number of such noncoding variants in the literature remains small, and because published epigenomic datasets also do not cover all tissues and cell states relevant to BMI. Future refinements to this integrative framework will likely enhance its predictive power as more functional validation data become available.

The inclusion of COVID-19 association as one of our seven prioritization criteria introduces an exploratory cross-trait metric with inherent limitations. Obesity is clinically linked to adverse COVID-19 progression. However, genomic overlap between BMI and COVID-19 GWAS signals does not establish a causal involvement in adipocyte biology or primary energy metabolism. Indeed, our observation of mixed protective and risk direction signals across the COVID-19 association sub-tracks argues against a simple or uniform mechanistic relationship between BMI and COVID-19 severity genetic associations. Therefore, the COVID-19 association criterion is presented solely as an exploratory associative context to highlight potential immuno-metabolic pleiotropy at loci carrying both BMI and COVID-19 signals.

Additionally, although our manual annotation protocol followed standardized criteria, features that rely on visual assessment of peak clarity or background thresholds, which in this analysis are largely limited to ENCODE histone modification ChIP-seq data, inevitably retain a degree of subjective judgment, which represents an inherent limitation regarding inter-observer reproducibility. We defined a clear, well-above background peak call as an ENCODE histone modification signal in the ENCODE Regulation track that was readily visually apparent as a peak flanked by regions of no signal (Supplementary Figure S2). Future implementations would benefit from transitioning toward automated, threshold-based algorithmic pipelines to standardize feature extraction across genome-scale datasets.

Future research should focus on direct functional validation of highest-scoring lncRNA, intronic, and intergenic SNPs, by siRNA knockdowns and overexpression of transcripts that contain or overlap the SNPs, and also by CRISPR-based genome editing, including knockouts and allele replacement. These studies should be conducted in adipocyte-relevant or other metabolically relevant primary cell models to allow for a definitive assessment of these lncRNAs’ roles in cellular processes governing BMI.

For rs2245368, we hypothesize that the variant may influence BMI-associated biology by altering the expression, splicing, or transcript stability of the *DTX2P1-UPK3BP1-PMS2P11* pseudogene-associated transcriptional unit in which it resides. This may be testable by allele-specific genome editing in adipocyte-relevant or other metabolically relevant human cell models, followed by assessment of transcript abundance, isoform usage, nearby gene expression, local chromatin features, and downstream cellular phenotypes. Such experimental data may also support refinement of our prioritization approach, including the development of biologically informed weighted scoring systems based on the relative functional contribution of different annotation features.

## 4. Materials and Methods

### 4.1. Manual Annotation

**Figure 3:**
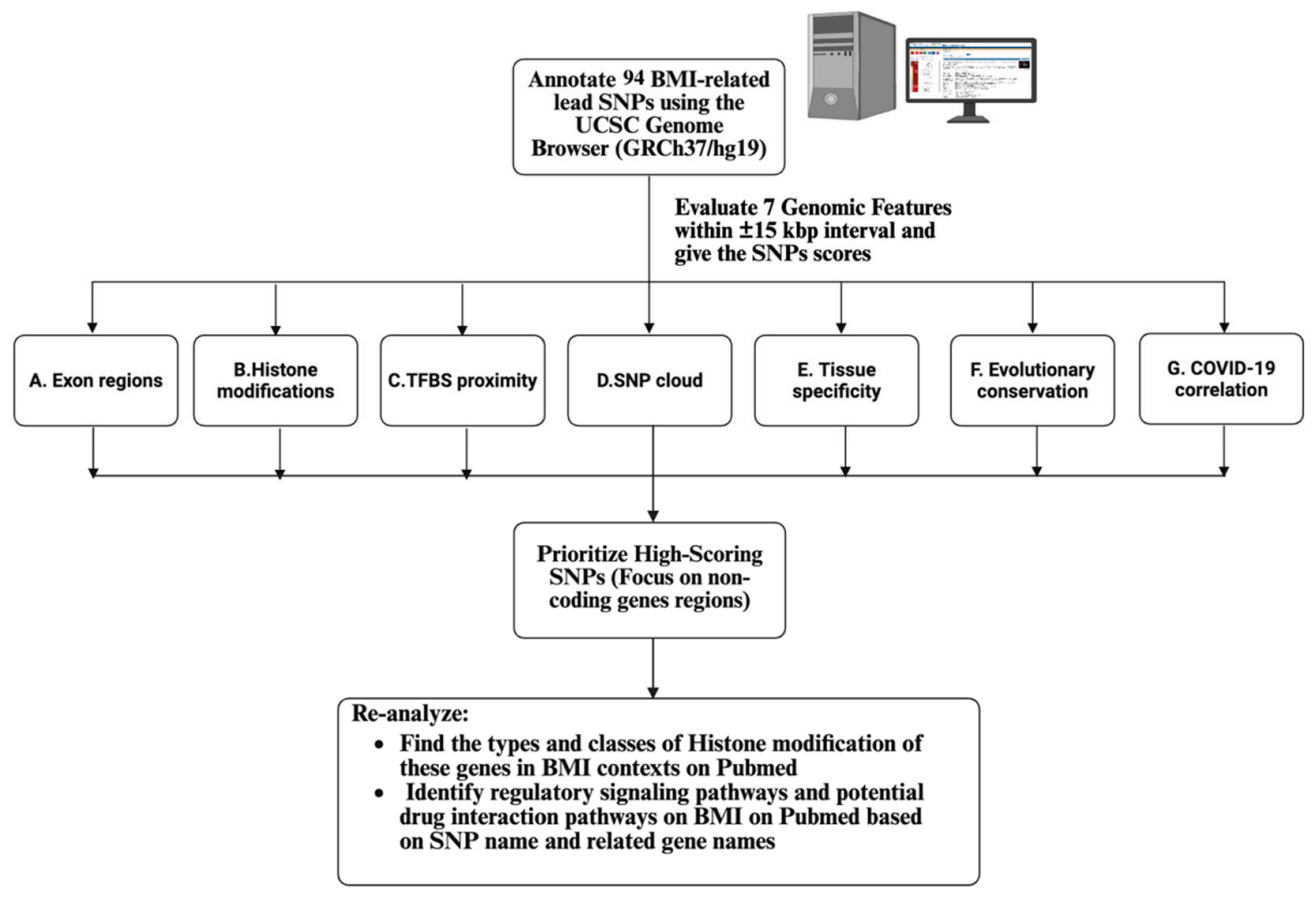
Workflow for prioritizing BMI-associated SNPs. Ninety-four lead SNPs were annotated in the UCSC Genome Browser (GRCh37/hg19). Each SNP was evaluated within a 501 bp interval (±250 bp) and an expanded 30 kb region (±15 kb) for seven functional criteria: gene localization (exonic, intronic, or intergenic), histone modifications (H3K4Me1, H3K4Me3, H3K27Ac), transcription factor binding sites, SNP clouds, tissue-specific expression, evolutionary conservation across vertebrates, and COVID-19 associations. This multicriteria scoring system prioritized SNPs, particularly those within long non-coding RNA genes, to identify high-priority candidates for causal follow-up studies targeting obesity-related regulatory mechanisms.

We annotated the lead SNPs for 94 loci associated with BMI [28] using the UCSC Genome Browser, GRCh37/hg19 human genome assembly version [65]. For each locus, we defined an initial annotation interval of 501 bp centered on the lead SNP (250 bp before, the SNP base, and 250 bp after) and then zoomed out to a total interval size of 30 kb, i.e., 15 kb on each side of the SNP, in order to annotate selected additional features not evident on the smaller initial scale. To assess each genetic variant’s source locus as a potential direct, causal disease candidate, we analyzed seven specific biological features (Figure 3): localization in gene exons, histone modifications, transcription factor binding sites, SNP clouds (defined later in this section), tissue expression and cell type specificity, evolutionary conservation, and genetic associations with COVID severity. These were independent of each other; therefore, one feature would not influence the other six. All collected data was current as of 30 April 2025.

#### 4.1.1. Feature I: Localization in Gene Exons

We used three gene annotation tracks in the UCSC Genome Browser to classify each SNP as exonic (with respect to any protein-coding or non-coding gene or transcript catalogued in these tracks), intronic, or intergenic. We utilized the UCSC Genes track, which combines data from NCBI RNA Reference Sequences (RefSeq), GenBank, Consensus CDS protein set (CCDS), and several other reference gene model catalogs [66]. We also utilized the NCBI RefSeq Gene track [67] because it is a commonly accepted annotation “gold standard.” Third, we used the GENCODE Genes track, version 47/September 2024 [8]. When annotating each SNP, we took all these tracks into consideration to determine whether it was exonic to a catalogued gene. In addition, we employed the “Genbank mRNA” and “human EST” tracks, repositories of highly accurate Sanger-sequenced transcriptomic data spanning 30 years of massive cDNA library sequencing efforts worldwide, to test for the presence of a transcriptional unit at each locus and the localization of the index SNP relative to its exons.

For all annotation criteria, we issued simple binary scores, one score per criterion. Each SNP was rated by the sum of its scores. For Feature I, a score of 1 was issued if the SNP was exonic to a protein-coding gene or a non-coding RNA transcript from any of the datasets listed above. When the SNP was intronic, it was given 0.5 points; finally, when a SNP was intergenic, it was scored at 0 points. The UCSC Genome Browser (hg19) was accessed from September 2024 until May 2025 (main US UCSC Genome Browser site) for all work discussed.

#### 4.1.2. Feature II: Histone Modifications

Histone modifications, including methylations and acetylations of specific amino acids, allow for decondensation of the chromatin, which facilitates gene expression [68]. A putative activating enhancer region can be indicated by a monomethylation (H3K4Me1) or acetylation (H3K27Ac) of histones, or especially, by the concomitant incidence of those two modifications. Hence, the area is denoted as a putative enhancer region if both signals are present. High levels of histone-3 lysine 4 trimethylation (H3K4Me3) indicate the presence of a putative promoter.

Each locus was examined for proximity to select histone modification signals, because they are indicative of promoter or enhancer elements and therefore can elucidate the function of a SNP-containing genomic region even in the absence of gene exons or introns covering the SNP. Accordingly, we screened each interval of ±15 kbp from the lead SNP for the presence of H3K4Me1 (regulatory element signature), H3K4Me3 (promoter signature), and H3K27Ac (active regulatory element signature). The rationale for selecting a ±15 kbp interval (a total window size centered on each lead SNP) was inspired by established functional paradigms of mammalian gene regulation, such as the human β-globin locus control region (LCR), where clustered regulatory elements span distances ranging from 6 to over 22 kb [69]. By extending beyond the immediate 1–2 kb proximal promoter region upstream of the transcription start site, this 15 kb window affords a better opportunity to capture multi-element local cis-regulatory landscapes—including nearby enhancers and locus control elements—that functionally interact with gene promoters. At the same time, this threshold intentionally avoids overly broad intervals (e.g., hundreds of kilobases) that are more likely to cross topologically associated domain (TAD) boundaries or linkage disequilibrium (LD) breakpoints, thereby minimizing the introduction of unrelated background noise.

To prevent false positives associated with the 15 kb window criterion, our prioritization framework includes multi-layered scoring. A high priority score is assigned only when an epigenetic mark within the 15 kb window is corroborated by localized, high-resolution functional features evaluated within a much stricter 501 bp window (250 bp) around the SNP, such as experimentally determined Transcription Factor Binding Site (TFBS) occupancy from ENCODE Consortium ChIP-seq data or the presence of a SNP cloud.

Histone modifications were inferred from chromatin immunoprecipitation followed by next-generation sequencing (ChIP-seq) data from ENCODE Consortium Tier 1 and Tier 2 cell lines [70]. We used the ENCODE Consortium Tier 1 and Tier 2 ChIP-seq datasets displayed in the “ENC Histone” supertrack of the UCSC Genome Browser (Histone Modifications from ChIP-seq from ENCODE/Broad, Stanford, U. Washington, etc.) and examined the three marks H3K4Me1, H3K4Me3, and H3K27Ac within the ±15 kb annotation window around each lead SNP.

For Feature II, a score of 1 was issued if the SNP was within 15 kbp of at least one of these three epigenetic modification types, provided that the modification resided in a clear and discrete, well-above-background ChIP-seq peak visible in manual UCSC Genome Browser-based annotation. Otherwise, a score of 0 was issued.

#### 4.1.3. Feature III: Transcription Factor Binding Sites

This feature considered whether the SNP was inside, or within 501 bp of, an experimentally supported transcription factor binding site (TFBS) [71,72]. Two subtracks of the ENCODE Regulation integrated track reported this information: Transcription Factor ChIP-seq from ENCODE (V3) and Transcription Factor (161 Factors) ChIP-seq from ENCODE with FactorBook. ENCODE (V3) incorporates 130 cell types, with data from 338 factors. Factorbook shows the consensus motifs of different TFs’ binding sites [73], based on 91 human cell lines [74]. These tracks contain experimental genomewide ChIP-seq datasets [75].

For Feature III, a score of 1 was issued if the SNP was within 501 bp of a TFBS ChIP-seq signal area (regardless of the cells or tissues of origin) visible during our manual UCSC Genome Browser-based annotation (Supplementary Table S1). TFBS ChIP-seq signals are marked as rectangles on their subtracks of the ENCODE Regulation track, hence there was no visual ambiguity during annotation. Otherwise, a score of 0 was issued.

#### 4.1.4. Feature IV: SNP Cloud

The NHGRI/EBI Catalog of Published Genome-Wide Association Studies track (“GWAS Variants,” i.e., the “green track”) of the UCSC Browser displays SNPs with strong, published, statistically significant associations with specific common disease phenotypes and/or specific quantitative traits [76]. We define a “SNP cloud” as a genomic interval containing at least 2 green-track SNPs significantly associated with any adiposity, obesity, or related cardiometabolic or anthropometric traits within a 501 bp region centered on the lead SNP. The SNPs comprising the “cloud” were required to be functionally relevant to each other (i.e., to be significantly associated with similar disease phenotypes) in order to meet this definition. For instance, three “GWAS catalog” track SNPs within a 501-bp interval that are all related to anthropometric traits (or three SNPs related to BMI, obesity, and diabetes) would be defined as comprising a SNP cloud. On the other hand, three “GWAS catalog” track SNPs clustered together within 501 bp but linked to three different and unrelated traits would not count as a SNP cloud. We examined each locus for the presence of a SNP cloud, recording the database identifier (rs number) and associated trait/s of each SNP in the cloud. For Feature IV, a score of 1 was issued if the SNP was within a SNP cloud as defined above. Otherwise, a score of 0 was issued for the SNP.

#### 4.1.5. Feature V: Tissue Expression and Cell Type Specificity

Another factor that was used in the evaluation of the index SNPs at these BMI loci was evidence of expression of genes in adiposity-relevant tissues, and, for non-genic loci, evidence of histone modifications and TFBSs that occurred in adiposity-relevant tissues or cell line models. The GTEx (Genotype-Tissue Expression) abundance profiles, as well as tissue-of-origin information for mRNAs and ESTs, give information on the tissue where a gene is expressed. We checked these expression profiles for all genes that harbored significant variants, as well as for all genes and transcriptional units located (partly or wholly) within 15 kbp of non-genic significant variants (Supplementary Table S1).

For Feature V, whenever the SNP was located in (exonic or intronic to) a coding or non-coding (including lncRNA) gene and the GTEx tissue track data were available for that gene, we scored the tissue-specific expression data from GTEx. A signal maximum level was defined as the top of the tissue’s expression box in the gene’s GTEx boxplot, not including the outliers above the box. If the gene or lncRNA corresponded to a signal maximum level exceeding 150 tags per million (TPM) in multiple tissues, the SNP score was 1.0. When a single tissue or cell type shows a signal maximum level exceeding 150 TPM, the score was 0.8. Among multiple tissues or cell types, the SNP score of GTEx associated with signal maximum between 15–150 TPM was 0.6, while the SNP score of having a single maximum within this range was 0.4. If the gene or lncRNA is associated with more than one tissue and the highest signal level was between 0.5 and 15 TPM, the score was 0.2. In the absence of any such association, the SNP’s score was 0. In the absence of GTEx data, expression evidence was inferred from cDNA or expressed sequence tags (ESTs). If cDNA or EST support is present, the score was 0.2; if both exist, the score increases to 0.4.

To assess the promoter activity of transcriptional units harboring or nearest to the SNPs, we utilized the FANTOM5 Consortium’s Cap Analysis of Gene Expression (CAGE) Phase1 “CTSS human tracks pooled filtered with 3 or more tags per library and rle normalized” dataset [39].This dataset provides CAGE-based promoter expression profiles normalized using the Relative Log Expression (RLE) method, with a filtering criterion of a minimum of three CAGE tags per library to ensure signal reliability. To ensure statistical power, we established a sample size threshold. If a cell line or tissue had fewer than five valid data points (i.e., tissues where CAGE-based expression at promoter regions met the filtering criteria) in this primary dataset, it was considered insufficient for robust analysis. In those cases, we employed a secondary data source: the FANTOM5 “CAGE Phase1 BAM human with Q3 filter and rle normalized” dataset. This dataset uses a more lenient Q3 (third quartile) filtering standard, designed to capture a greater number of low-abundance transcription start events.

#### 4.1.6. Feature VI: Evolutionary Conservation

The Conservation Track of the UCSC Genome Browser displays whole-genome multiple sequence alignments across 100 vertebrate species, along with quantitative measures of evolutionary conservation derived by two methods (phastCons and phyloP) from the Phylogenetic Analysis with Space/Time models (PHAST) suite of tools [77]. We assessed the evolutionary significance of potential SNPs by manually zooming in to a range of 51 bps (25 bps left, 1 bp SNP, 25 bps right) or less to compare the conservation of individual bases across different species. To integrate sequence data from ancient humans and provide a context for interpreting the nonhuman primate alignments to the human genome, we also visualized the Neandertal Seq (full) and Denisovan Seq (full) tracks of the UCSC Genome Browser to document the major alleles, at the location orthologous to each human SNP, in those two species of ancient humans. Coding, and functional, sequences are more conserved, and this becomes more apparent as the genetic distance decreases [78]. However, the conservation of gene structures, as opposed to that of gene sequences, was not routinely examined prior to our foundational study [79]. We analyzed the conservation of key gene structure elements (KGSEs), including the splice sites at the start and the end of each intron (GT/AG, respectively) and of the polyadenylation signal consensus sequence near the 3′end of the last exon (AATAAA or ATTAAA). For each KGSE, we recorded the closest non-human species in which the KGSE was not conserved relative to humans, and the most distant non-human species in which the KGSE was conserved. For Feature VI, a score of 1 was given if the SNP was located within a region of high evolutionary conservation, with conserved bases across the vast majority of 100 vertebrate species, particularly non-human primates. Otherwise, a score of 0 was issued.

#### 4.1.7. Feature VII: COVID Associations

We considered the COVID GWAS v4 track, which presents the fourth GWAS Data Release (October 2020) of the COVID-19 Host Genetics Initiative (HGI). This initiative aimed to identify the genetic factors influencing susceptibility to SARS-CoV-2 infection, as well as the severity and outcomes of the disease. The output color within the track displays the GWAS effect: red denotes deleterious and blue denotes protective. The height of the items reflects the statistical significance, with the default setting showing only SNPs with *p* < 0.001. The intensity of the color indicates the magnitude of the effect, with more vivid colors representing effects above the median and paler colors representing effects below the median. We examined four subtracks for COVID: A2(Severe COVID variants/“vars”), B2(Hospitalization COVID vars), C1(Tested COVID vars), C2(All COVID vars), to assist in judging the value of SNPs [15]. This feature was included because severe COVID-19 outcomes are positively correlated with obesity and elevated BMI. Since poor COVID-19 results are widely recognized to be associated with metabolic and inflammatory pathways shared with obesity, COVID-19 severity serves as a legitimate biological correlate and associative measure of BMI-related biology [16].

For Feature VII, a score was assigned based on the association with COVID as presented in the track. A score of 0.5 was issued if the SNP was extensively associated with COVID-19 (at least any two of the four subtracks), and an additional 0.5 was added if the intensity of the color indicated a magnitude of effect above the median. The scores for Features I through VII were then added to generate a single combined score for each SNP, ranging from 0 to 7. Accordingly, as one of the seven primary scoring criteria in our integrated framework, this feature provides a legitimate associative measure of BMI-associated loci based on clinical relevance observed during the pandemic.

#### 4.1.8. Extension of the Annotation Region for Top-Scoring SNPs

Subsequently, for all SNPs with total scores of 5 or greater, we performed a second round of UCSC Genome Browser-based manual annotation within a wider region centered on the SNP, in order to understand whether the global genomic landscape of each region, beyond the 30 kbp intervals considered during the first round of annotation, contained additional epigenetic or expression features pointing to functional, candidate causal non-coding variants in proximity to the lead SNP. There was no set maximum extended interval. We stopped zooming out in the UCSC Genome Browser whenever we detected additional functional features.

### 4.2. Competing Endogenous RNA (ceRNA) Network Analysis

#### 4.2.1. Prediction of lncRNA–miRNA Interactions

Predicted interactions between lncRNAs and miRNAs were obtained from the LncBase v3 database [80]. To ensure reliability, the following filters were applied: (i) Biotypes were restricted to lincRNA, antisense, and processed transcript; (ii) Species was limited to *Homo sapiens*; (iii) Experimental support included high-confidence methods such as high-throughput sequencing of RNAs isolated by crosslinking immunoprecipitation (HITS-CLIP), photoactivatable ribonucleoside-enhanced crosslinking and immunoprecipitation (PAR-CLIP), argonaute immunoprecipitation (AGO-IP), luciferase reporter assay, chimeric fragments, covalent ligation of endogenous Argonaute-bound RNA-crosslinking immunoprecipitation (CLEAR-CLIP), miR-CLIP, and RNA immunoprecipitation followed by quantitative PCR (RIP-qPCR); (iv) Confidence level was set to high; (v) Tissue sources included brain, liver, liver bile duct, blood, bone, bone marrow, lymph nodes, nervous system, pancreas, muscle, ventricle, and peripheral blood. These criteria were designed to maximize the selection of experimentally supported and biologically relevant lncRNA–miRNA interactions.

#### 4.2.2. Prediction of miRNA–mRNA Interactions

Predicted mRNA targets were retrieved from TargetScan [81,82]. We applied thresholds on context score (≤−0.20) and P_CT (≥0.20), and considered only strong seed match types (8mer and 7mer-m8), in line with established miRNA–mRNA prediction strategies [83,84].

#### 4.2.3. Tissue Co-Expression Filtering

To increase biological relevance, lncRNA–miRNA and miRNA–mRNA pairs were further filtered based on tissue co-expression in humans. First, expression of lncRNAs and mRNAs across human tissues was obtained from the Bgee database (https://www.bgee.org/), and only genes with an expression score > 60 were retained [85]. Second, lncRNA and mRNA expression data was retrieved from the GTEx database (https://gtexportal.org/home/), and transcripts with TPM ≥ 1 in human tissues were considered expressed [86]. For each predicted interaction, the lncRNA, miRNA, and mRNA were required to be co-expressed within at least one tissue according to Bgee and GTEx. Only triplets meeting this tissue co-expression criterion were preserved for downstream ceRNA network construction.

#### 4.2.4. Network Visualization and Analysis

The lncRNA–miRNA–mRNA triplets were integrated into a competing endogenous RNA (ceRNA) network. The resulting interactions were exported as edge tables and imported into Cytoscape (version 9.0) for visualization [87].

## 5. Conclusions

Our comprehensive functional annotation of 94 BMI-associated SNPs suggests putative candidate non-coding regulatory elements in obesity genetics, with 91 of 94 variants residing in non-coding regions, including six within lncRNA genes and functionally significant intergenic and intronic SNPs. These variants cluster in tissue-specific regulatory elements active in adipose tissue, liver, and brain, supporting coordinated regulatory networks underlying complex metabolic traits. The intersection with COVID-19 susceptibility pathways provides exploratory context for potential underscores their pleiotropic effects. This study establishes an integrative framework for prioritizing GWAS variants based on functional evidence, pointing to testable new lncRNA and regulatory candidate targets for precision medicine in obesity. We establish a precedent for bridging the gap between statistical association signals and testable functional hypotheses through the inclusion of non-coding genes, regulatory elements, and evolutionary signatures that are often overlooked in conventional GWAS annotations.

## Data Availability

The original contributions presented in this study are included in the article. Further inquiries can be directed to the corresponding author(s).

## Supplementary Materials

The following supporting information can be downloaded at: https://www.mdpi.com/article/10.3390/ijms27157015/s1.

## References

[1] Maurano M.T., Humbert R., Rynes E., Thurman R.E., Haugen E., Wang H., Reynolds A.P., Sandstrom R., Qu H., Brody J. (2012). Systematic localization of common disease-associated variation in regulatory DNA. Science.

[2] Gregory T.R., Gregory T.R. (2005). CHAPTER 1—Genome Size Evolution in Animals. The Evolution of the Genome.

[3] Shkurat T.P., Ammar M., Bocharova O., Teplyakova E., Aleksandrova A., Ali R., Lipovich L. (2023). The Role of Genetic Variants in the Long Non-Coding RNA Genes MALAT1 and H19 in the Pathogenesis of Childhood Obesity. Noncoding RNA.

[4] Suhre K., McCarthy M.I., Schwenk J.M. (2021). Genetics meets proteomics: Perspectives for large population-based studies. Nat. Rev. Genet..

[5] Morris J.A., Caragine C., Daniloski Z., Domingo J., Barry T., Lu L., Davis K., Ziosi M., Glinos D.A., Hao S. (2023). Discovery of target genes and pathways at GWAS loci by pooled single-cell CRISPR screens. Science.

[6] Uffelmann E., Huang Q.Q., Munung N.S., de Vries J., Okada Y., Martin A.R., Martin H.C., Lappalainen T., Posthuma D. (2021). Genome-wide association studies. Nat. Rev. Methods Primers.

[7] Gholami M., Zoughi M., Larijani B., Amoli M., Bastami M. (2020). An in silico approach to identify and prioritize miRNAs target sites polymorphisms in colorectal cancer and obesity. Cancer Med..

[8] Mudge J.M., Carbonell-Sala S., Diekhans M., Martinez J.G., Hunt T., Jungreis I., Loveland J.E., Arnan C., Barnes I., Bennett R. (2025). GENCODE 2025: Reference gene annotation for human and mouse. Nucleic Acids Res..

[9] Moore J.E., Purcaro M.J., Pratt H.E., Epstein C.B., Shoresh N., Adrian J., Kawli T., Davis C.A., Dobin A. (2020). Expanded encyclopaedias of DNA elements in the human and mouse genomes. Nature.

[10] Katayama S., Tomaru Y., Kasukawa T., Waki K., Nakanishi M., Nakamura M., Nishida H., Yap C.C., Suzuki M., Kawai J. (2005). Antisense transcription in the mammalian transcriptome. Science.

[11] Engstrom P.G., Suzuki H., Ninomiya N., Akalin A., Sessa L., Lavorgna G., Brozzi A., Luzi L., Tan S.L., Yang L. (2006). Complex Loci in human and mouse genomes. PLoS Genet..

[12] Poupon-Bejuit L., Hughes M.P., Liu W., Geard A., Faour-Slika N., Whaler S., Massaro G., Rahim A.A. (2022). A GLP1 receptor agonist diabetes drug ameliorates neurodegeneration in a mouse model of infantile neurometabolic disease. Sci. Rep..

[13] Chen S., Gan D., Lin S., Zhong Y., Chen M., Zou X., Shao Z., Xiao G. (2022). Metformin in aging and aging-related diseases: Clinical applications and relevant mechanisms. Theranostics.

[14] Conte C., Cipponeri E., Roden M. (2024). Diabetes Mellitus, Energy Metabolism, and COVID-19. Endocr. Rev..

[15] Norouzi M., Norouzi S., Ruggiero A., Khan M.S., Myers S., Kavanagh K., Vemuri R. (2021). Type-2 Diabetes as a Risk Factor for Severe COVID-19 Infection. Microorganisms.

[16] Maddaloni E., Buzzetti R. (2020). Covid-19 and diabetes mellitus: Unveiling the interaction of two pandemics. Diabetes Metab. Res. Rev..

[17] Liu B., Du Y., Wu Y., Snetselaar L.G., Wallace R.B., Bao W. (2021). Trends in obesity and adiposity measures by race or ethnicity among adults in the United States 2011-18: Population based study. BMJ.

[18] Bays H.E., Toth P.P., Kris-Etherton P.M., Abate N., Aronne L.J., Brown W.V., Gonzalez-Campoy J.M., Jones S.R., Kumar R., La Forge R. (2013). Obesity, adiposity, and dyslipidemia: A consensus statement from the National Lipid Association. J. Clin. Lipidol..

[19] Kosmas C.E., Rodriguez Polanco S., Bousvarou M.D., Papakonstantinou E.J., Peña Genao E., Guzman E., Kostara C.E. (2023). The triglyceride/high-density lipoprotein cholesterol (TG/HDL-C) ratio as a risk marker for metabolic syndrome and cardiovascular disease. Diagnostics.

[20] Putra I., Daly M., Sutin A., Steptoe A., Robinson E. (2023). The psychological legacy of past obesity and early mortality: Evidence from two longitudinal studies. BMC Med..

[21] Bouchard C. (2021). Genetics of Obesity: What We Have Learned Over Decades of Research. Obesity.

[22] Loos R.J.F., Yeo G.S.H. (2022). The genetics of obesity: From discovery to biology. Nat. Rev. Genet..

[23] GBD Adult BMI Collaborators (2025). Global, regional, and national prevalence of adult overweight and obesity, 1990–2021, with forecasts to 2050: A forecasting study for the Global Burden of Disease Study 2021. Lancet.

[24] Dall T.M., Sapra T., Natale Z., Livingston T., Chen F. (2024). Assessing the economic impact of obesity and overweight on employers: Identifying opportunities to improve work force health and well-being. Nutr. Diabetes.

[25] NCD Risk Factor Collaboration (2024). Worldwide trends in underweight and obesity from 1990 to 2022: A pooled analysis of 3663 population-representative studies with 222 million children, adolescents, and adults. Lancet.

[26] Zhang W., Xu J., Li J., Guo T., Jiang D., Feng X., Ma X., He L., Wu W., Yin M. (2018). The TEA domain family transcription factor TEAD4 represses murine adipogenesis by recruiting the cofactors VGLL4 and CtBP2 into a transcriptional complex. J. Biol. Chem..

[27] Wang Y., Beydoun M.A., Min J., Xue H., Kaminsky L.A., Cheskin L.J. (2020). Has the prevalence of overweight, obesity and central obesity levelled off in the United States? Trends, patterns, disparities, and future projections for the obesity epidemic. Int. J. Epidemiol..

[28] Locke A.E., Kahali B., Berndt S.I., Justice A.E., Pers T.H., Day F.R., Powell C., Vedantam S., Buchkovich M.L., Yang J. (2015). Genetic studies of body mass index yield new insights for obesity biology. Nature.

[29] Hemerich D., Svenstrup V., Obrero V.D., Preuss M., Moscati A., Hirschhorn J.N., Loos R.J.F. (2024). An integrative framework to prioritize genes in more than 500 loci associated with body mass index. Am. J. Hum. Genet..

[30] Ndiaye F.K., Huyvaert M., Ortalli A., Canouil M., Lecoeur C., Verbanck M., Lobbens S., Khamis A., Marselli L., Marchetti P. (2020). The expression of genes in top obesity-associated loci is enriched in insula and substantia nigra brain regions involved in addiction and reward. Int. J. Obes..

[31] Saeed S., Bonnefond A., Froguel P. (2025). Obesity: Exploring its connection to brain function through genetic and genomic perspectives. Mol. Psychiatry.

[32] The ENCODE Project Consortium (2012). An integrated encyclopedia of DNA elements in the human genome. Nature.

[33] Kent W.J., Sugnet C.W., Furey T.S., Roskin K.M., Pringle T.H., Zahler A.M., Haussler D. (2002). The human genome browser at UCSC. Genome Res..

[34] Manning A.K., Goustin A.S., Kleinbrink E.L., Thepsuwan P., Cai J., Ju D., Leong A., Udler M.S., Brown J.B., Goodarzi M.O. (2020). A Long Non-coding RNA, LOC157273, Is an Effector Transcript at the Chromosome 8p23.1-PPP1R3B Metabolic Traits and Type 2 Diabetes Risk Locus. Front. Genet..

[35] Zhao J., Zhang W., Lin M., Wu W., Jiang P., Tou E., Xue M., Richards A., Jourd’heuil D., Asif A. (2016). MYOSLID Is a Novel Serum Response Factor-Dependent Long Noncoding RNA That Amplifies the Vascular Smooth Muscle Differentiation Program. Arter. Thromb. Vasc. Biol..

[36] Liu P., Huang S., Ling S., Xu S., Wang F., Zhang W., Zhou R., He L., Xia X., Yao Z. (2019). Foxp1 controls brown/beige adipocyte differentiation and thermogenesis through regulating β3-AR desensitization. Nat. Commun..

[37] Tong Q., Dalgin G., Xu H., Ting C.N., Leiden J.M., Hotamisligil G.S. (2000). Function of GATA transcription factors in preadipocyte-adipocyte transition. Science.

[38] Estell C., Davidson L., Steketee P.C., Monier A., West S. (2021). ZC3H4 restricts non-coding transcription in human cells. eLife.

[39] FANTOM Consortium (2014). A promoter-level mammalian expression atlas. Nature.

[40] Hon C.-C., Ramilowski J.A., Harshbarger J., Bertin N., Rackham O.J., Gough J., Denisenko E., Schmeier S., Poulsen T.M., Severin J. (2017). An atlas of human long non-coding RNAs with accurate 5’ ends. Nature.

[41] McGonagle D., Sharif K., O’Regan A., Bridgewood C. (2020). The Role of Cytokines including Interleukin-6 in COVID-19 induced Pneumonia and Macrophage Activation Syndrome-Like Disease. Autoimmun. Rev..

[42] Gao H., Hamp T., Ede J., Schraiber J.G., McRae J., Singer-Berk M., Yang Y., Dietrich A.S., Fiziev P.P., Kuderna L.F. (2023). The landscape of tolerated genetic variation in humans and primates. Science.

[43] Biglari S., Youssefian L., Tabatabaiefar M.A., Saeidian A.H., Abtahi-Naeini B., Khorram E., Sherkat R., Moghaddam A.S., Mohaghegh F., Rahimi M. (2025). DOCK2 Deficiency and GATA2 Haploinsufficiency Can Underlie Critical Coronavirus Disease 2019 (COVID-19) Pneumonia. J. Clin. Immunol..

[44] Menghini R., Marchetti V., Cardellini M., Hribal M.L., Mauriello A., Lauro D., Sbraccia P., Lauro R., Federici M. (2005). Phosphorylation of GATA2 by Akt increases adipose tissue differentiation and reduces adipose tissue-related inflammation: A novel pathway linking obesity to atherosclerosis. Circulation.

[45] Adam M., Imboden M., Schaffner E., Boes E., Kronenberg F., Pons M., Bettschart R., Barthelemy J.C., Schindler C., Probst-Hensch N. (2017). The adverse impact of obesity on heart rate variability is modified by a NFE2L2 gene variant: The SAPALDIA cohort. Int. J. Cardiol..

[46] Park B., Chang S., Lee G.J., Kang B., Kim J.K., Park H. (2019). Wnt3a disrupts GR-TEAD4-PPARγ2 positive circuits and cytoskeletal rearrangement in a β-catenin-dependent manner during early adipogenesis. Cell Death Dis..

[47] Salmena L., Poliseno L., Tay Y., Kats L., Pandolfi P.P. (2011). A ceRNA hypothesis: The Rosetta Stone of a hidden RNA language?. Cell.

[48] Claussnitzer M., Dankel S.N., Kim K.H., Quon G., Meuleman W., Haugen C., Glunk V., Sousa I.S., Beaudry J.L., Puviindran V. (2015). FTO Obesity Variant Circuitry and Adipocyte Browning in Humans. N. Engl. J. Med..

[49] Freedman M.L., Monteiro A.N., Gayther S.A., Coetzee G.A., Risch A., Plass C., Casey G., De Biasi M., Carlson C., Duggan D. (2011). Principles for the post-GWAS functional characterization of cancer risk loci. Nat. Genet..

[50] Weng S., Fu H., Xu S., Li J. (2024). Validating core therapeutic targets for osteoporosis treatment based on integrating network pharmacology and informatics. SLAS Technol..

[51] Lou W., Ding B., Fu P. (2020). Pseudogene-Derived lncRNAs and Their miRNA Sponging Mechanism in Human Cancer. Front. Cell Dev. Biol..

[52] Rana S., Bhatti A.A. (2021). Predicting anthropometric and metabolic traits with a genetic risk score for obesity in a sample of Pakistanis. Sci. Rep..

[53] Yoo A., Lee S. (2023). Neuronal growth regulator 1 may modulate interleukin-6 signaling in adipocytes. Front. Mol. Biosci..

[54] Gordon A.C., Mouncey P.R., Al-Beidh F., Rowan K.M., Nichol A.D., Arabi Y.M., Annane D., Beane A., van Bentum-Puijk W., Berry L.R. (2021). Interleukin-6 Receptor Antagonists in Critically Ill Patients with Covid-19. N. Engl. J. Med..

[55] Ando W., Horii T., Uematsu T., Hanaki H., Atsuda K., Otori K. (2021). Impact of overlapping risks of type 2 diabetes and obesity on coronavirus disease severity in the United States. Sci. Rep..

[56] Minster R.L., Hawley N.L., Su C.-T., Sun G., Kershaw E.E., Cheng H., Buhule O.D., Lin J., Reupena M.a.S., Viali S.i. (2016). A thrifty variant in CREBRF strongly influences body mass index in Samoans. Nat. Genet..

[57] Benton M.L., Abraham A., LaBella A.L., Abbot P., Rokas A., Capra J.A. (2021). The influence of evolutionary history on human health and disease. Nat. Rev. Genet..

[58] Neel J.V. (1962). Diabetes mellitus: A “thrifty” genotype rendered detrimental by “progress”?. Am. J. Hum. Genet..

[59] Speakman J.R. (2008). Thrifty genes for obesity, an attractive but flawed idea, and an alternative perspective: The ‘drifty gene’ hypothesis. Int. J. Obes..

[60] Hughes G.J. (2022). The Thrifty Gene Hypothesis: A Total Review. Bachelor’s Thesis.

[61] Zhang H., Yu L., Chen J., Liu L., Yang X., Cui H., Yue G. (2022). Role of Metabolic Reprogramming of Long non-coding RNA in Clear Cell Renal Cell Carcinoma. J. Cancer.

[62] Li M., Xie Z., Wang P., Li J., Liu W., Tang S., Liu Z., Wu X., Wu Y., Shen H. (2018). The long noncoding RNA GAS5 negatively regulates the adipogenic differentiation of MSCs by modulating the miR-18a/CTGF axis as a ceRNA. Cell Death Dis..

[63] Sun X., Lin J., Zhang Y., Kang S., Belkin N., Wara A.K., Icli B., Hamburg N.M., Li D., Feinberg M.W. (2016). MicroRNA-181b Improves Glucose Homeostasis and Insulin Sensitivity by Regulating Endothelial Function in White Adipose Tissue. Circ. Res..

[64] Bowker N., Shah R.L., Sharp S.J., Luan J., Stewart I.D., Wheeler E., Ferreira M.A.R., Baras A., Wareham N.J., Langenberg C. (2020). Meta-analysis investigating the role of interleukin-6 mediated inflammation in type 2 diabetes. eBioMedicine.

[65] Nassar L.R., Barber G.P., Benet-Pagès A., Casper J., Clawson H., Diekhans M., Fischer C., Gonzalez J.N., Hinrichs A.S., Lee B.T. (2023). The UCSC genome browser database: 2023 update. Nucleic Acids Res..

[66] Hsu F., Kent W.J., Clawson H., Kuhn R.M., Diekhans M., Haussler D. (2006). The UCSC Known Genes. Bioinformatics.

[67] Goldfarb T., Kodali V.K., Pujar S., Brover V., Robbertse B., Farrell C.M., Oh D.-H., Astashyn A., Ermolaeva O., Haddad D. (2025). NCBI RefSeq: Reference sequence standards through 25 years of curation and annotation. Nucleic Acids Res..

[68] Gagnidze K., Pfaff D.W. (2022). Epigenetic mechanisms: DNA methylation and histone protein modification. Neuroscience in the 21st Century: From Basic to Clinical.

[69] Levings P.P., Bungert J. (2002). The human beta-globin locus control region. Eur. J. Biochem..

[70] Hoffman M.M., Ernst J., Wilder S.P., Kundaje A., Harris R.S., Libbrecht M., Giardine B., Ellenbogen P.M., Bilmes J.A., Birney E. (2013). Integrative annotation of chromatin elements from ENCODE data. Nucleic Acids Res..

[71] Rigor P. (2015). Applications in High-Throughput Sequencing Technologies: From Compression Algorithms and Data Warehousing to Understanding Gene Regulation and Diseases at Scale. Doctoral Dissertation.

[72] Statkiewicz M., Maryan N., Kulecka M., Kuklinska U., Ostrowski J., Mikula M. (2019). Functional analyses of a low-penetrance risk variant rs6702619/1p21. 2 associating with colorectal cancer in Polish population. Acta Biochim. Pol..

[73] Wang J., Zhuang J., Iyer S., Lin X.Y., Greven M.C., Kim B.H., Moore J., Pierce B.G., Dong X., Virgil D. (2013). Factorbook.org: A Wiki-based database for transcription factor-binding data generated by the ENCODE consortium. Nucleic Acids Res..

[74] Wang J., Zhuang J., Iyer S., Lin X., Whitfield T.W., Greven M.C., Pierce B.G., Dong X., Kundaje A., Cheng Y. (2012). Sequence features and chromatin structure around the genomic regions bound by 119 human transcription factors. Genome Res..

[75] Gerstein M.B., Kundaje A., Hariharan M., Landt S.G., Yan K.K., Cheng C., Mu X.J., Khurana E., Rozowsky J., Alexander R. (2012). Architecture of the human regulatory network derived from ENCODE data. Nature.

[76] Sollis E., Mosaku A., Abid A., Buniello A., Cerezo M., Gil L., Groza T., Güneş O., Hall P., Hayhurst J. (2023). The NHGRI-EBI GWAS Catalog: Knowledgebase and deposition resource. Nucleic Acids Res..

[77] Raney B.J., Barber G.P., Benet-Pages A., Casper J., Clawson H., Cline M.S., Diekhans M., Fischer C., Navarro Gonzalez J., Hickey G. (2024). The UCSC Genome Browser database: 2024 update. Nucleic Acids Res..

[78] Leypold N.A., Speicher M.R. (2021). Evolutionary conservation in noncoding genomic regions. Trends Genet..

[79] Wood E.J., Chin-Inmanu K., Jia H., Lipovich L. (2013). Sense-antisense gene pairs: Sequence, transcription, and structure are not conserved between human and mouse. Front. Genet..

[80] Karagkouni D., Paraskevopoulou M.D., Tastsoglou S., Skoufos G., Karavangeli A., Pierros V., Zacharopoulou E., Hatzigeorgiou A.G. (2020). DIANA-LncBase v3: Indexing experimentally supported miRNA targets on non-coding transcripts. Nucleic Acids Res..

[81] Agarwal V., Bell G.W., Nam J.-W., Bartel D.P. (2015). Predicting effective microRNA target sites in mammalian mRNAs. Elife.

[82] Lewis B.P., Burge C.B., Bartel D.P. (2005). Conserved seed pairing, often flanked by adenosines, indicates that thousands of human genes are microRNA targets. Cell.

[83] Friedman R.C., Farh K.K.-H., Burge C.B., Bartel D.P. (2009). Most mammalian mRNAs are conserved targets of microRNAs. Genome Res..

[84] Grimson A., Farh K.K.-H., Johnston W.K., Garrett-Engele P., Lim L.P., Bartel D.P. (2007). MicroRNA targeting specificity in mammals: Determinants beyond seed pairing. Mol. Cell.

[85] Bastian F.B., Roux J., Niknejad A., Comte A., Fonseca Costa S.S., de Farias T.M., Moretti S., Parmentier G., de Laval V.R., Rosikiewicz M. (2021). The Bgee suite: Integrated curated expression atlas and comparative transcriptomics in animals. Nucleic Acids Res..

[86] GTEx Consortium (2020). The GTEx Consortium atlas of genetic regulatory effects across human tissues. Science.

[87] Shannon P., Markiel A., Ozier O., Baliga N.S., Wang J.T., Ramage D., Amin N., Schwikowski B., Ideker T. (2003). Cytoscape: A software environment for integrated models of biomolecular interaction networks. Genome Res..

