# Supplementary material for: Functional Annotation of GWAS Loci Using Public Transcriptome and Epigenome Datasets Reveals Non-Coding Genes and Regulatory Elements Which May Contribute to BMI
Source: Int J Mol Sci. 2026 Aug 4;27(15):7015. doi: 10.3390/ijms27157015 (PMC13466785; doi:10.3390/ijms27157015)
Supplement: Supplementary file 1 [file ijms-27-07015-s001.zip › Supplementary Figures S1 and S2.pdf]

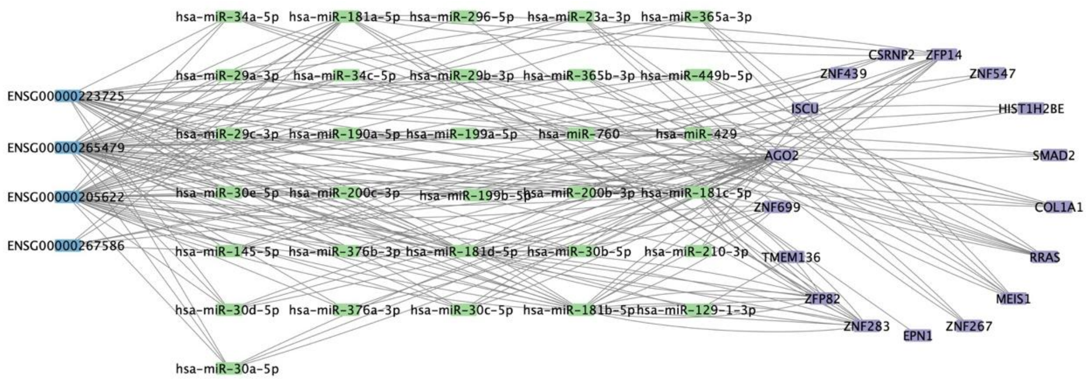

Supplementary Figure S1. Tissue-filtered ceRNA network based on Bgee expression data. LncRNAs, miRNAs, and mRNAs were retained only if their expression scores exceeded 60 in at least one common human tissue.

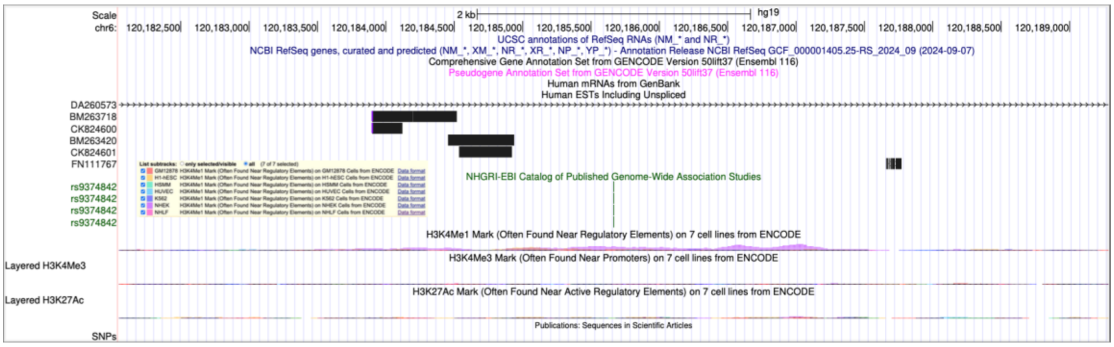

Supplementary Figure S2. Overlap of an ENCODE histone modification ChIP-seq peak without a BMI-associated SNP from our input dataset.
